# [Expression of Concern] Radiosensitization of esophageal carcinoma cells by knockdown of RNF2 expression

**DOI:** 10.3892/ijo.2025.5818

**Published:** 2025-11-10

**Authors:** Xing-Xiao Yang, Ming Ma, Mei-Xiang Sang, Xue-Xiao Wang, Heng Song, Zhi-Kun Liu, Shu-Chai Zhu

Int J Oncol 48: 1985-1996, 2016; DOI: 10.3892/ijo.2016.3404

Following the publication of the above paper, it was drawn to the Editor's attention by a concerned reader that certain of the westen blot data included in Fig. 4C and D looked strikingly similar, such that the same data may have been included more than once in these figure parts to show the results of differently performed experiments. Upon performing an independent analysis of the data in the Editorial Office, it also came to light that data featured in Fig. 3A of the above paper had been re-used in a figure in a paper featuring some of the same authors that was published in the journal *Scientific Reports*.

The authors were contacted by the Editorial Office to offer an explanation for this possible anomaly in the presentation of the data in this paper, although up to this time, no response from them has been forthcoming. Owing to the fact that the Editorial Office has been made aware of potential issues surrounding the scientific integrity of this paper, we are issuing an Expression of Concern to notify readers of this potential problem while the Editorial Office continues to investigate this matter further.

